# Simulation of Radiation Damage for Silicon Drift Detector

**DOI:** 10.3390/s19081767

**Published:** 2019-04-13

**Authors:** Yang Liu, Tengfei Zhu, Jianxi Yao, Xiaoping Ouyang

**Affiliations:** 1 School of Nuclear Science and Engineering, North China Electric Power University, Beijing 102206, China; 1162212036@ncepu.edu.cn (T.Z.); oyxp2003@aliyun.com (X.O.); 2 State Key Laboratory of Intense Pulsed Radiation Simulation and Effect, Xi’an 710024, China; 3 Renewable Energy School, North China Electric Power University, Beijing 102206, China; jianxiyao@ncepu.edu.cn

**Keywords:** silicon drift detector, radiation damage, displacement, ionization

## Abstract

Silicon drift detector with high sensitivity and energy resolution is an advanced detector which is suitable to be used in deep space detection. To study and reveal the radiation damage of the silicon drift detector (SDD) in a deep-space environment, which will degrade the detector performance, in this paper, the SDD radiation damage effects and mechanics, including displacement damage and ionization damage, for irradiations of different energy of neutrons and gammas are investigated using Geant4 simulation. The results indicate the recoil atoms distribution generated by neutrons in SDD is uniform, and recoil atoms’ energy is mainly in the low energy region. For secondary particles produced by neutron irradiation, a large energy loss in inelastic scattering and fission reactions occur, and neutron has a significant nuclear reaction. The energy deposition caused by gammas irradiation is linear with the thickness of SDD; the secondary electron energy distribution produced by gamma irradiation is from several eV to incident particle energy. As the scattering angle of secondary electron increases, the number of secondary electrons decreases. Therefore, a reasonable detector epitaxial thickness should be set in the anti-irradiation design of SDD.

## 1. Introduction

In the field of deep-space exploration, X-ray navigation [1] and X-ray communication [2] are two further revolutionary concepts proposed by National Aeronautics and Space Administration in 2015 [3]; the development of their technology has strategy significance worldwide. Due to the weak signal of pulsar and complex space environment, X-ray navigation and X-ray communication require the development of X-ray radiation detectors which have high energy resolution and time resolution. Silicon drift detectors (SDDs) were proposed in 1983 by Gatti and Rehak [4] as high-resolution position-sensitive detectors for fast-ionizing particles and spectroscopy of X-rays. In recent years, because the technology has become matured in terms of material growth and device fabrication, SDDs are widely used in different application [5,6,7,8], especially in deep-space exploration, because SDD has preeminent properties such as high sensitivity, high energy resolution, low leakage current, and high quantum efficiency. So, it is suitable to be used in X-ray navigation and X-ray communication as a preferable substitution for conventional radiation detectors such as microchannel plate and focused detector.

In deep space, it is well known that the performance of any silicon-based detector and its energy resolution degrades due to the environmental effects as well as radiation damage. SDD, as a special silicon-based detector, is sensitive to displacement damage which is produced by the non-ionizing energy loss of charged and neutral particles. The displacement damage leads to an increase in detector leakage current and thus degrades the energy resolution. So, the radiation damage is the primary cause of output shifts and function breakdown, so it is important to study radiation damage for SDDs produced by the various charged and neutral particles with various energy. Many experimental studies of radiation damage have been conducted. Change of leakage current and charge-collection efficiency in large area SDDs with proton spectrum centered at 11.2 MeV for a fluence of 10^9^/cm^2^ are reported [9,10]. Radiation measurements on various types of silicon detectors have been reported with protons [11,12], gamma rays [13,14], and neutrons [15,16].

However, previous experimental studies for radiation damage silicon-based detectors including SDDs usually focus on a specific particle type with specific energy, then experimental results of detector characteristics are obtained. On one hand, the practical working environment of SDDs in deep space is a complex environment full of various particles with different energy; it differs from the experimental environments, and obviously the practical detector performance characteristics in a practical complex space environment is not a simple superposition of the characteristics under the sole-particle radiation field. On the other hand, it is important to reveal radiation damage mechanism of SDDs to find optimal strategies to reduce irradiation damage before employing them in specific application. So, it is required to simulate SDD performance under a complex deep-space environment, especially simulating the radiation damage under neutron and gamma particles that could occur on SDD with high penetrability because of their electric neutrality. Their energies are deposited in the SDD via two mechanisms of atomic collisions and electronic ionization, producing displacement damage and ionization damage, respectively. Under the exposure of these electrically neutral particles, the performances of SDD may be reduced by either ionization or displacement damage. SDD is sensitive to both ionizing and displacement effects induced by neutrons and gammas. So, radiation damage effects induced by neutrons and gammas on SDD, in general, are important from both basic and applied points of view. It is instructive to reveal the damage of SDD due to displacement damage and ionization damage.

Radiation damage mechanisms, including simulations in silicon, have been studied for many years. In previous research, they usually focus on the displacement damage, point defect, cluster defect, nonionizing energy loss [17,18,19,20,21], and the energy and species of primary knock-on atom (PKA) [22,23]. However, there is no research focusing on the spatial distribution, scattering angle, and energy spectrum of the PKA, and other secondary particles, such as, gammas, protons, alphas, and neutrons. The energy distribution, scattering angle, and spatial distribution of the PKA produced by the neutrons and other secondary particles are of great significance for the study of the evolutionary process of detector irradiation defects. The purpose of this paper is to research the mechanism of displacement damage and ionization damage in SDD caused by neutrons and gammas using Geant4 simulation. Geant4 is a toolkit for the simulation of the passage of particles through matter. Its areas of application include high-energy, nuclear, and accelerator physics, as well as studies in medical and space science [24]. In the present work, attention has been focused on the study of radiation damage produced by different energy neutrons in SDDs, and the results are compared with gammas.

## 2. Radiation Damage

When neutrons of sufficient energy irradiate SDD, one or more heavy recoils called primary knock-on atom (PKA) can be formed. The displacement damage in SDD caused by neutrons is primarily due to displacing a PKA out of its lattice site. If the energy of PKA is bigger than the threshold energy of lattice atom, the PKA will continue to collide with the recoil atom, leading to recoil atoms called secondary knock-on atom (SKA). When recoil atoms collide in SDD, it will cause ionizing energy deposition, which is equal to the product of trajectory distance and ionization cross section. Recoil atoms continue to collide with lattice atoms, which cause the nonionizing energy deposition that conclude vacancies and interstitials [25]. The recoil atom will not be tracked until it passes through the material or its energy is less than the cutoff value.

The nonionizing energy deposition (NIEL) caused by neutrons is neutron energy deposition colliding with nuclei, including elastic energy loss, inelastic energy loss, and coulomb energy loss. NIEL is defined as follows

(1) $$NIEL(T_{0})=\frac{N_{A}}{A}\int_{T_{\min}}^{T_{\max}}TQ(T){\left(\frac{d\sigma }{dT}\right)}_{T_{0}}dT,$$

where *N_A_* is Avogadro constant, *A* is atomic number of material, *T_max_* and *T_min_* are maximum and minimum energy of primary displaced atoms, and *dσ/dT* is differential cross section of displacement damage. *Q(T)* is the function of a dislocated atom’s energy, which represents the energy loss caused by inelastic collision and elastic collision of displaced atoms. In the simulation, NIEL is usually calculated by the following formula:

(2) $$NIEL(T)=\frac{N_{A}}{A}\sigma_{d}E_{d}=\frac{N_{A}}{A}\frac{E_{d}}{N_{\nu }x}=\frac{E_{d}}{\rho x}=\frac{TQ(T)}{\rho x},$$

where *σ_d_* is the displacement damage section, *E_d_* is the displacement loss energy, *N_v_* is the atomic density of target atoms, x is the target thickness, and *ρ* is the target density.

The scattering angle can be obtained by two-body elastic scattering. With the scattering angle, we can obtain the energy and direction of the recoil atom. In the case of elastic scattering of neutrons, the recoil energy is given by

(3) $$E_{out}=\frac{4AE_{in}}{{\left(1+A\right)}^{2}}\cos^{2}u,$$

where *E_in_* and *E_out_* are the incident and output neutron energy, and *u* is the scattering angle of recoil relative to the direction of the incoming neutron.

**Figure 1:**
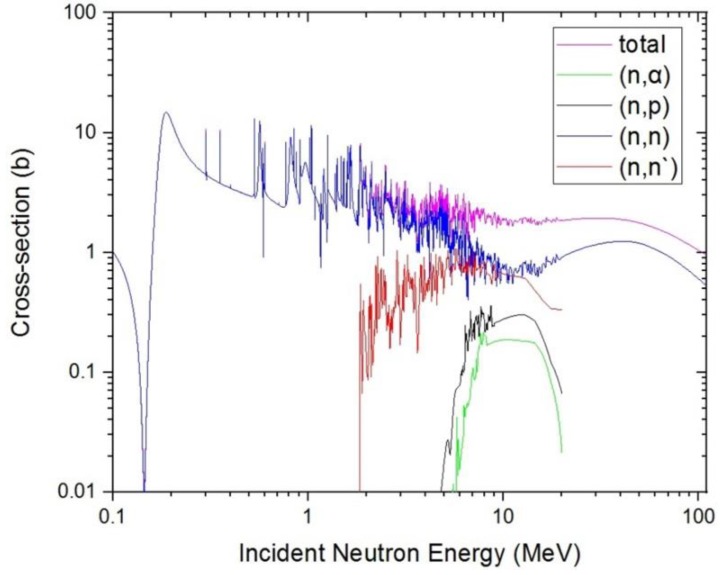
The nuclear reaction cross-section database of neutrons and Si.

There are different physical processes when neutrons transport in materials. The cross-section of different physical processes is closely related to the energy of neutrons, in order to accurately analyze the subsequent simulation results. According to the neutron reaction cross-section database of RSCII, the relationship between the neutron energy and the main reaction cross sections of Si is plotted as shown in Figure 1.

Gamma photons are electrically neutral particles like neutrons and they have a strong penetrability. Radiation damage caused by gamma photons in SDD is mainly ionization damage, and the displacement damage is very small. The ionizing energy deposition of 1 MeV gamma photons is substantially equal to the ionizing energy deposition of 1 MeV electron in semiconductor device. Although gamma photons can directly collide with lattice atom in SDD, the energy transmitted to the nucleus is only a few dozen eV, which is substantially equal to the lattice element’s deviation threshold energy, since the probability of gamma photons collided with nucleus is very small. In the analysis of radiation damage mechanism caused by gamma photons in semiconductor devices, it requires considering the gamma photon energy.

The Klein–Nishina formula gives the differential cross section of photons scattered from a single free electron in the lowest order of quantum electrodynamic. For an incident photon of energy $E_{\gamma }$, the differential cross section is:

(4) $$\frac{d\sigma }{d\Omega }=\alpha^{2}r_{c}^{2}P{\left(E_{r},\theta \right)}^{2}[P(E_{r},\theta )+P{\left(E_{r},\theta \right)}^{-1}-1+\cos^{2}(\theta )]/2,$$

where $\frac{d\sigma }{d\Omega }$ is a differential cross section, dΩ is an infinitesimal solid angle element, α is the fine structure constant, θ is the scattering angle, $r_{c}=\hbar /m_{e}c$ is the “reduced” Compton wave length of the electron, $m_{e}$ is the mass of an electron, and $P(E_{r},\theta )$ is the ratio of photon energy after and before the collision:

(5) $$P(E_{r},\theta )=\frac{1}{1+(E_{\gamma }/m_{e}c^{2}0(1-\cos\theta )}=\frac{\lambda }{\lambda^{\prime }}.$$

## 3. Simulation and Validation

### 3.1. Simuation Model

**Figure 2:**
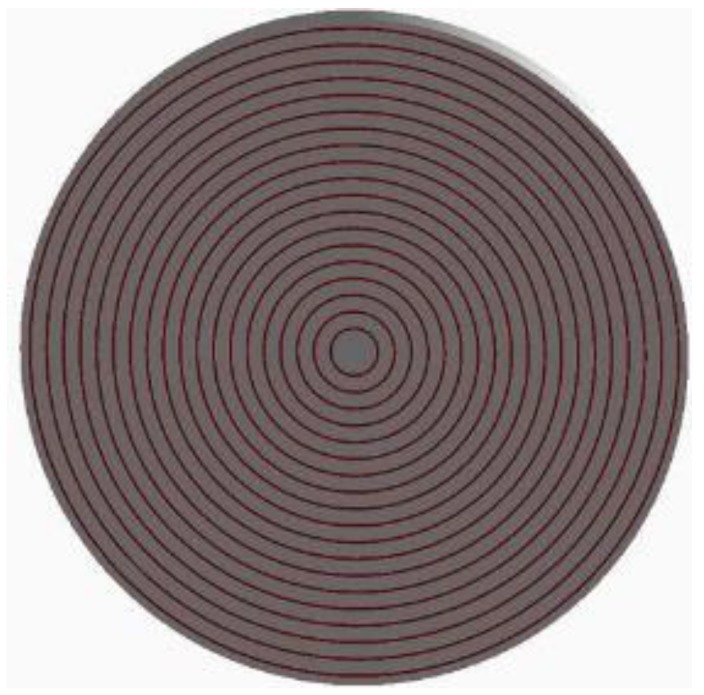
Physical model of silicon drift detector (SDD) using Geant4.

In the field of deep-space radiation detection, especially X-ray detection application for silicon-based detectors such as SDDs, there are neutrons with wide energy of 10 keV–20 MeV and high-energy gamma rays in the deep-space environment. When neutrons irradiate SDD, displacement damage effect leads to defects in SDD, the periodic potential field around lattice is destroyed, then defect energy levels are induced in bandgap of semiconductor material which decay the carrier density, lifetime, mobility, and charge-collection efficiency of SDD. When high-energy gamma rays irradiate SDD, ionizing energy deposition (IEL) induces instant light current and positive charges on grid electrode of SDD, which reduce the threshold voltage of detector. In our research group, we are now fabricating SDD, focusing on X-ray detection in deep space; taking account of the material quality and size and level of device fabrication technique, we design the SDD as silicon substrate covered by SiO_2_ drift ring with concentric ring structure. We selected the geometry parameters as follows: The radium of silicon substrate is 1 cm and thickness is 500 μm. The thickness of SiO_2_ drift ring is 60 nm and the width of each ring is 25 μm. In the simulation, we have used Geant4 version 10.3 for our simulations. As a physics list, we have chosen the QGSP_BIC_HP precompiled model, which is suitable for neutron processes with energies 0.025 eV–20 MeV and can simulate the secondary particle transport process well. The precompiled model contains G4LCapture, G4NeutronHPCature, G4LFisson, G4NeutronHPFission, G4LENeutronInelastic, G4LEastic, and G4NeutronHPElastic. When gammas irradiate the SDD, three primary interactions occur: Compton scattering, photoelectric effect, and pair production. The key to statistical sampling of secondary particles for these physical processes is to establish appropriate energy transfer and scattering cross sections. So, we selected G4LivemorePhotoElectricModel, G4LivemorePhotoComptonModel, and G4G4LivemoreGammaConversionModel. The physical model of SDD using Geant4 is shown in Figure 2.

### 3.2. Radiation Damage Caused by Neutrons in SDD

#### 3.2.1. Displacement Damage

- Track structure

**Figure 3:**
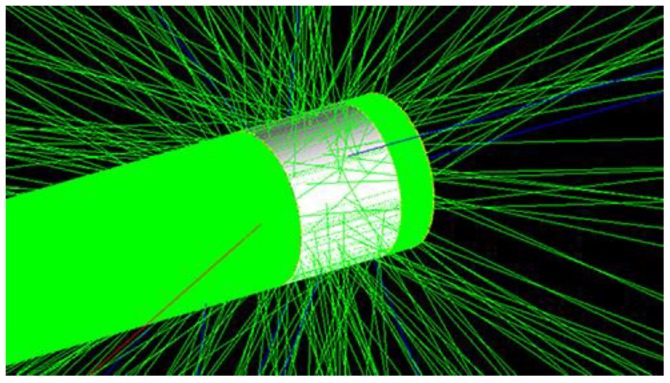
Track structure produced by neutrons irradiating SDD.

We simulate the track of neutrons irradiating SDD using Geant4 as shown in Figure 3 and we can see the majority of neutrons directly penetrate SDD, and just the minority of neutrons have been scattered. The most scattering angles are small angles, which belong to forward scattering. In the figure, green indicates electro-neutral particles, red indicates negative-point particles, and blue indicates positively charged particles. The green portion is mainly neutrons.

- PKA spatial distribution

**Figure 4:**
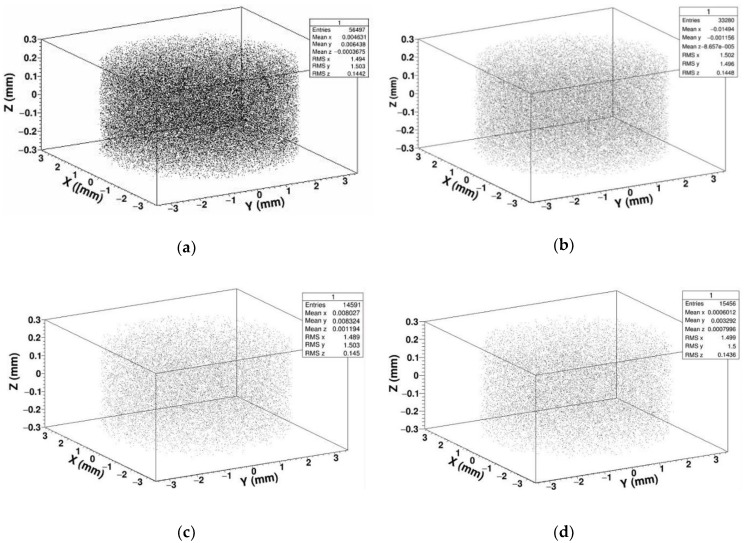
Primary knock-on atom (PKA) spatial distribution in SDD generated by neutrons: (**a**) PKA spatial distribution for 1 MeV neutrons; (**b**) PKA spatial distribution for 2 MeV neutrons; (**c**) PKA spatial distribution for 14 MeV neutrons; (**d**) PKA spatial distribution for 20 MeV neutrons.

In order to study the displacement damage caused by neutrons irradiating SDD, we calculated and extracted the spatial coordinates of PKA generated by different energy of neutrons, and reconstructed the spatial coordinates in three dimensions, forming the PKA spatial distribution shown in Figure 4.

As shown in the above figures, we simulated 5 × 10^6^ neutrons with different energy irradiating SDD which generate tens of thousands of PKA. In the three-dimensional reconstruction of PKA, we can see the position of PKA is almost evenly distributed in SDD. This is because the thickness of SDD, which is applied for soft X-ray detection in deep space, is usually designed to be several hundred micrometers. When high-energy neutrons irradiate SDD, the thickness of SDD is much lower than the mean free path of neutrons; that is to say all the neutrons in SDD collide almost randomly, if a neutron collided in SDD, the time of its collision is just one. Therefore, the probability of producing PKA at each position of SDD is even.

- PKA energy spectrum

**Figure 5:**
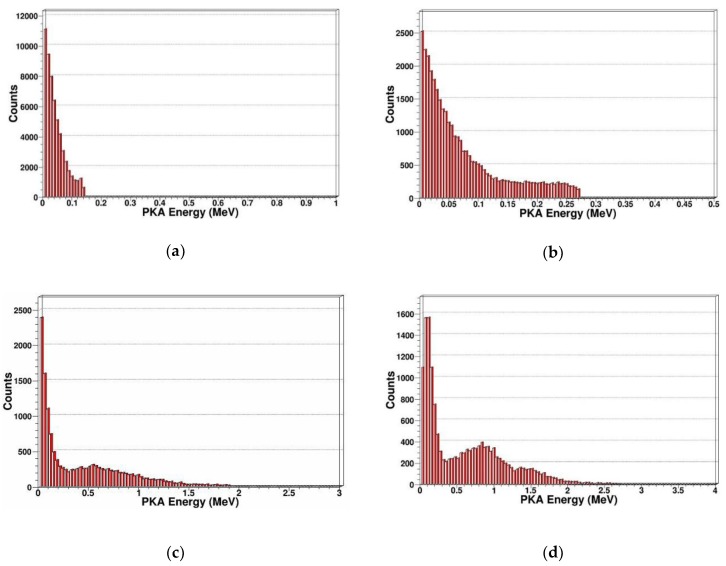
PKA energy spectrum in SDD generated by neutrons: (**a**) PKA energy spectrum for 1 MeV neutrons; (**b**) PKA energy spectrum for 2 MeV neutrons; (**c**) PKA energy spectrum for 14 MeV neutrons; (**d**) PKA energy spectrum for 20 MeV neutrons.

Recoil atoms play an important role in radiation damage; In SDD, different incident particle energy or different incident particle type can produce different energy spectrums of recoil atoms. Therefore, we calculated and extracted the PKA energy spectrums that were produced by neutrons with different energy as shown in Figure 5.

From Figure 5, we can see for specific neutron energy, with the PKA energy increasing, the number of PKA decreases. For 1 MeV, 2 MeV, 14 MeV, and 20 MeV neutrons, the maximum energy of PKA increase are 0.13 MeV, 0.26 MeV, 1.9 MeV, and 2.7 MeV, respectively. For 14 MeV and 20 MeV neutrons, there is a peak at about 0.65 MeV and 0.85 MeV in the energy spectrum. This is because the PKAs are created by elastic and inelastic collisions, when the energy of incidence neutron is not very high, as shown in Figure 5a,b, the elastic collision is predominant, and the energy spectrum of the emitting neutron and recoil atom after elastic collision is continuous and monotonous. When the energy of incidence neutron is higher than several MeV, as shown in Figure 5c,d, the probability of inelastic collision increases, then the resonant inelastic effects occur, which correspond to the peak in energy spectrum of the emitting neutron and PKA. In the above figures, we can see the PKA are mainly concentrated in the low-energy region, and mainly occur in low-energy transmission events. Some high-energy PKA will continue to generate SKA by cascade collision.

- PKA scatting angle

**Figure 6:**
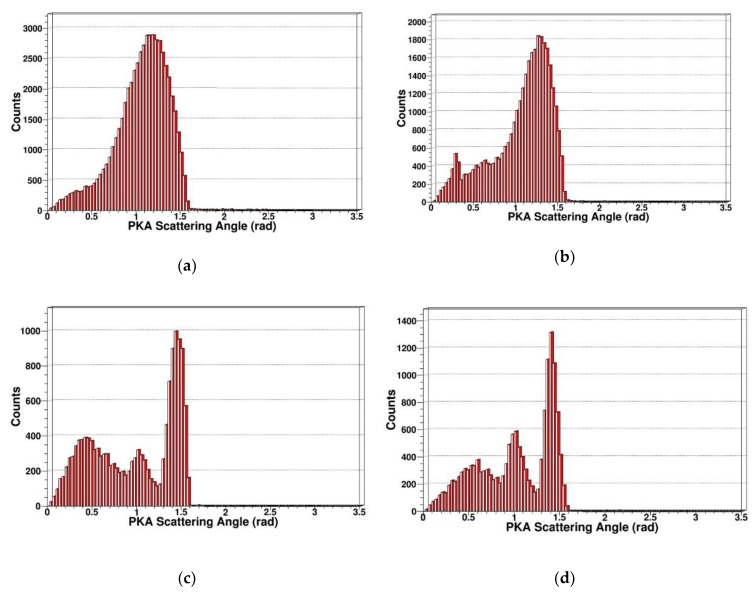
PKA scatting angle in SDD generated by neutrons: (**a**) PKA scatting angle for 1 MeV neutrons; (**b**) PKA scatting angle for 2 MeV neutrons; (**c**) PKA scatting angle for 14 MeV neutrons; (**d**) PKA scatting angle for 20 MeV neutrons.

In order to further analyze the PKA, we simulated and extracted the scattering angle distribution of PKA, which was generated by neutrons with different energy, as shown in Figure 6.

From the above figures, we can see the PKA scattering angles are concentrated in the range of 0 to 90 degrees, which belongs to forward scattering, and have a very obvious directionality. When the energy of incidence neutron is not very high, the elastic collision is predominant. When the energy of incidence neutron is higher than several MeV, the probability of inelastic collision increases, then the resonant inelastic effects occur and induce the scattering angle distribution, which is not monotonous, as shown in Figure 6c,d.

- Secondary knock-on atom

**Figure 7:**
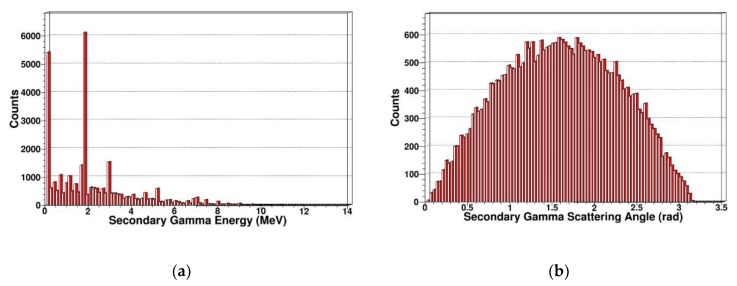
Secondary gammas in SDD generated by neutrons: (**a**) Energy spectrum of secondary gammas; (**b**) scattering angle distribution of secondary gammas.

**Figure 8:**
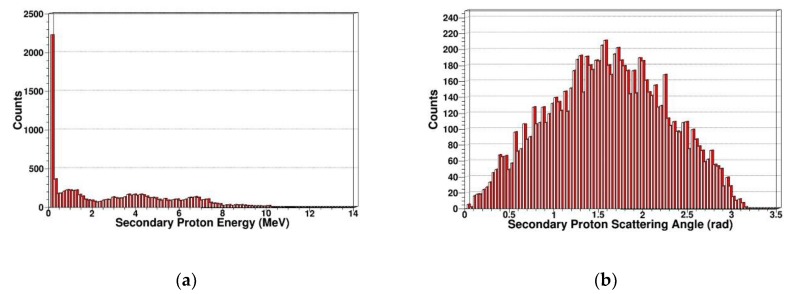
Secondary protons in SDD generated by neutrons: (**a**) Energy spectrum of secondary protons; (**b**) scattering angle distribution of secondary protons.

**Figure 9:**
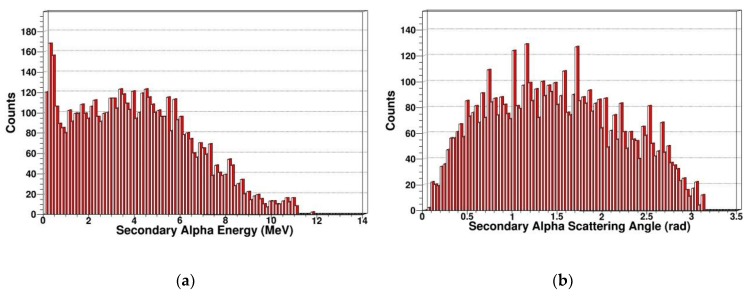
Secondary alphas in SDD generated by neutrons: (**a**) Energy spectrum of secondary alphas; (**b**) scattering angle distribution of secondary alphas.

**Figure 10:**
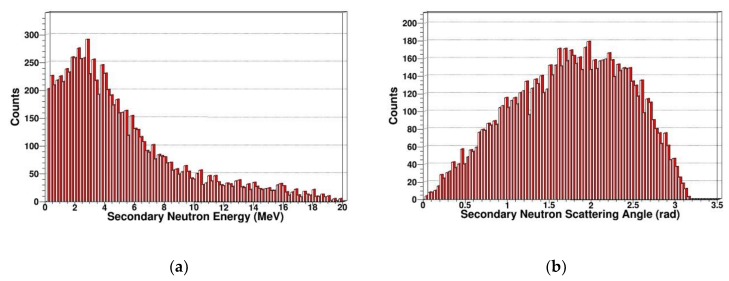
Secondary neutrons in SDD generated by neutrons: (**a**) Energy spectrum of secondary neutrons; (**b**) scattering angle distribution of secondary neutrons.

The neutron is an electrically neutral particle, which cannot cause ionization damage. But the neutron can produce multiple reactions including elastic scattering, inelastic scattering, radiation capture, and fission reaction in SDD. PKA is generated by elastic scattering. We simulated and extracted the energy spectrum and scattering angle of secondary gammas, secondary protons, secondary alphas, and secondary neutrons. In Figure 7, Figure 8, Figure 9 and Figure 10, some sharp peaks occur in the distribution; they are real resonances induced by certain resonant reaction channel, but the detailed physical mechanics and processes are complicated.

As shown in Figure 7, we simulated the energy spectrum and scattering angle distribution of secondary gammas in SDD generated by neutrons. It indicates that the secondary gammas are mainly concentrated in low-energy regions, where the maximum energy has reached 9.8 MeV, and the number of secondary gammas is relatively large. The energy loss of inelastic scattering in which neutrons occurred in SDD is relatively large, which corresponds to the reaction cross section of inelastic scattering in Figure 1. The scattering angle, which is approximately Gaussian, is distributed in at 0–180 degrees.

As shown in Figure 8, we simulated the energy spectrum and scattering angle distribution of secondary gammas in SDD generated by protons. It indicates that when neutrons irradiate, SDD produced a lot of secondary protons, which is on the same order of magnitude as the secondary gammas, and some secondary protons are high-energy particles. Neutrons occur in an obviously nuclear reaction in SDD, which corresponds to the reaction cross section of (n, p) in Figure 1. The scattering angle, which is approximately Gaussian, is distributed in at 0–180 degrees.

As shown in Figure 9, compared with secondary protons and secondary gammas, the order of magnitude of secondary alpha is much smaller. With the secondary alpha’s energy increasing, the number of secondary alphas increases first and then decreases, reaching a maximum at around 4 MeV and a maximum energy of alpha particles around 11.6 MeV. It indicates that the energy loss of neutrons is relatively large in fission reaction, which corresponds to the reaction cross section of (n, α) in Figure 1. The scattering angle, which is approximately Gaussian, is distributed at 0–180 degrees.

As shown in Figure 10, we can see there are many high-energy secondary neutrons in SDD generated by neutrons, and the number of secondary neutrons is relatively large. It is shown that the neutron has an obvious nuclear reaction in SDD, which corresponds to the reaction cross section of (n, n) in Figure 1.

#### 3.2.2. Vacancy Defect

The atomic deviation threshold of crystal is related to the material and the collision direction, and it forms the offset threshold section with the lattice direction. Evaluating the radiation damage intensity caused by incident particles can be based on the number of recoil atoms generated by incident particles. According to the total flux of incident particles, energy spectrum, and offset threshold section, we calculated the number of recoil atoms and the energy spectrum of recoil atoms in the differential energy spectrum. Then, we built a relation between the number of offsite atoms generated by recoil atoms with *E*, which is the basis to calculate the number of off-site atoms.

According to Norgett-Robinson-Torrens model [26], *N_d_*, which is the number of offsite atoms produced by PKA, can be defined as:

(6) $$\begin{array}{l} N_{d}(E)=\left\{ \begin{array}{cc} 0, & E<E_{d}\\ 1, & E_{d}\leq E<2.5E_{d}\\ \frac{0.4E_{D}(E)}{E_{d}}, & E\geq 2.5E_{L} \end{array}\right. \end{array}$$

In Equation (6), *E_D_* represents the damage energy of PKA, which is defined by:

(7) $$E_{D}(E)=\frac{E}{1+k_{d}g(T/E_{d})}.$$

In our simulation using Geant4, this continuous process is generally considered to be separated steps, from emitting incident particles to interacting with the lattice atoms in SDD, until the energy is exhausted. Without considering the secondary particles generated by PKA, the number of offsite atoms produced by the incident particles in this step can be calculated according to the following formula:

(8) $$N_{d,step}=0.4(E_{D}(E_{kin,pre}-E_{D}(E_{kin,post}))/E_{d},$$

where *E_D_(E_kin,pre_)* and *E_D_(E_kin,post_)* represent the energy loss caused by each of the two points before and after the secondary particles are not produced. 

**Figure 11:**
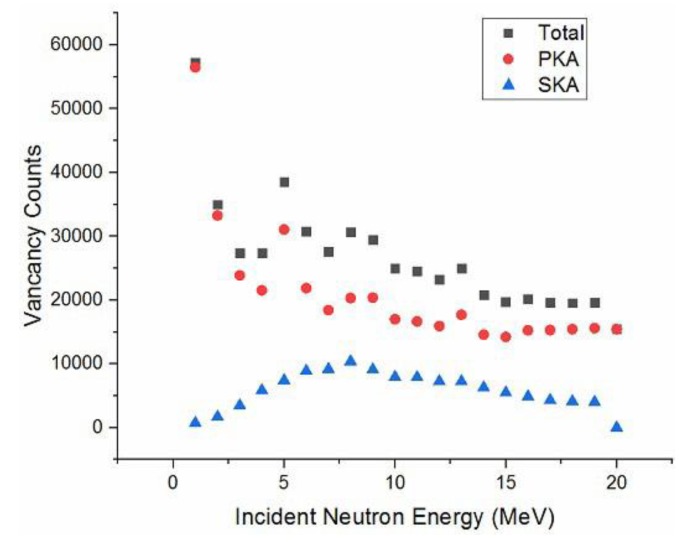
Relationship between the energy of neutrons and the number of defects.

In our simulation, SDD is irradiated by neutrons with different energy, which is at 1–20 MeV, and the number of off-site atoms and hole vacancy can be counted. In the study of the distribution of defects caused by neutrons in the SDD, the detector established above could be used. The relationship between incident energy of neutrons and the generation of hole vacancies is obtained by the calculation shown in Figure 11. We can see the change in trend of PKA is consistent with the total number of recoil atoms.

#### 3.2.3. Relationship between NIEL, IEL, and Thickness of SDD

When PKAs transport in SDD, some of the surrounding electrons can be formed to peel off, which causes PKAs to become charged particles. The effective charge of PKAs is related to the transport speed. The higher the transport speed is, the more extra nuclear electrons are stripped, and the more effective charge is generated. When charged particles collide with nucleus in SDD, the low energy mainly performed the elastic scattering. In our simulation calculation of non-ionizing energy deposition, we used the single-scattering to calculate. When PKAs interact with the electrons of target material in SDD, the energy loss is continuous during transport process. That is, the ionization energy deposition is equal to the product of the ionization blocking section and the track length. When PKAs collide with the nucleus, the energy loss is discrete. Meanwhile, part of the energy of PKA is transferred to the nucleus. Since the thickness of SDD is much smaller than the mean free path of incident particles, the ionization energy deposition and the non-ionization energy deposition are approximately linear with the thickness of the SDD.

**Figure 12:**
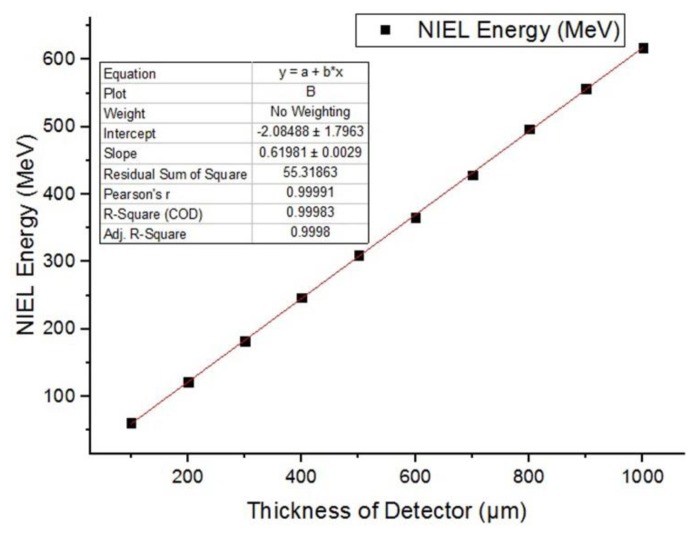
Relationship between nonionizing energy deposition (NIEL) produced by neutrons and the thickness of SDD.

**Figure 13:**
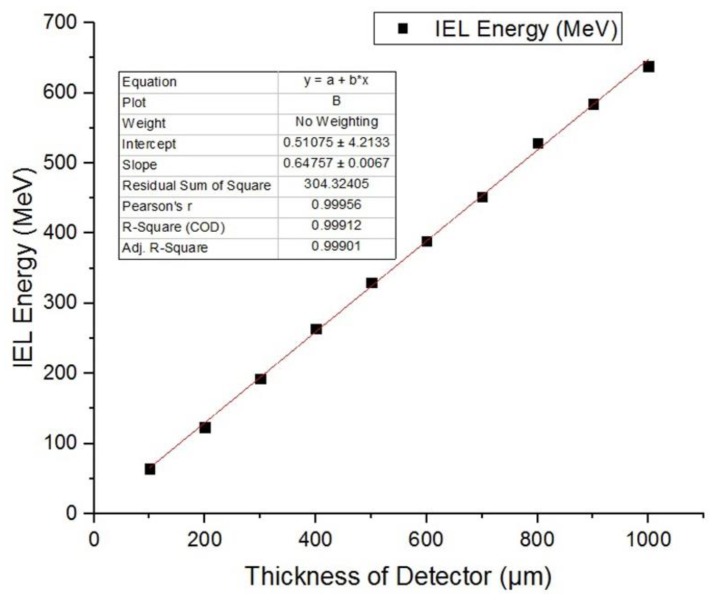
Relationship between ionizing energy deposition (IEL) produced by neutrons and the thickness of SDD.

In our simulation, 1 MeV neutrons were incident with a number of 10^6^. The thickness of SDD can be changed by changing the thickness of silicon substrate and counting the ionization energy deposition and the non-ionizing energy deposition. We selected the thickness of SDD to be 100 μm, 200 μm, 300 μm, 400 μm, 500 μm, 600 μm, 700 μm, 800 μm, 900 μm, and 1000 μm. The ionization energy deposition and non-ionizing energy deposition were recorded and fitted. The fitting results are as follows in Figure 12 and Figure 13.

From the above figures, we can see that the nonionizing energy deposition and ionizing energy deposition are approximately linear with the thickness of the SDD. The reason is that the mean free path of neutron is much larger than the geometric size of SDD. Fitting the IEL and NIEL curves in the above figures, we can obtain the coordinate of intersection between the curve and axis of detector thickness, which are (5.67, 0) for IEL curve, and (6.24, 0) for NIEL curve. The coordinate is not (0, 0), which is not because of the simulation error; that implies that when the detector thickness is lower than 5.67 μm, the IEL of neutrons in SDD is 0, and when the detector thickness is lower than 6.24 μm, the NIEL of neutrons in SDD is 0. Therefore, considering both the anti-irradiation reinforcement design and detection property of SDD, it is necessary to design a reasonable detector epitaxial thickness, which can capture the energy deposition of the target particle and reduce radiation damage caused by neutrons.

### 3.3. Radiation Damage Caused by Gammas in SDD

#### 3.3.1. Displacement Damage

- Track structure

**Figure 14:**
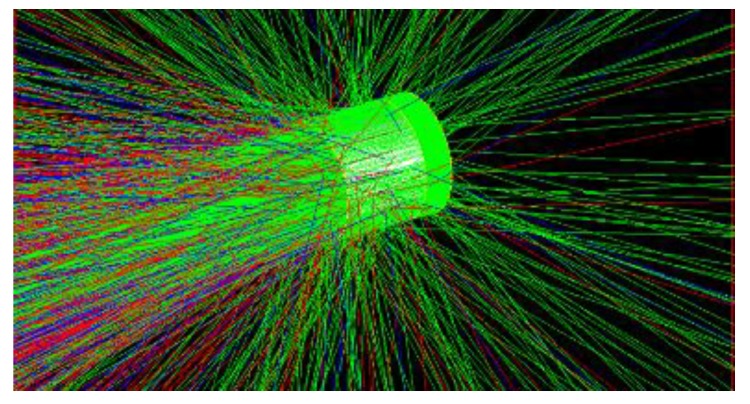
Track structure produced by gammas irradiating SDD.

We simulate the track of different energy of gammas irradiating SDD using Geant4 as shown in Figure 14, where red represents negatively charged particles, blue represents positively charged particles, and green represents electrically neutral particles. Our simulation of track structure indicates that most gamma photons directly penetrate SDD, most particles are scattered at small angles, and a minority of particles have large-angle scattering. Gamma photons generate a lot of charged particles in SDD. In the figure, green indicates electro-neutral particles, red indicates negative-point particles, and blue indicates positively charged particles. The green portion is mainly gammas, the blue portion is mainly positrons, and the red portion is mainly negative electrons.

- Primary knock-on atom

**Figure 15:**
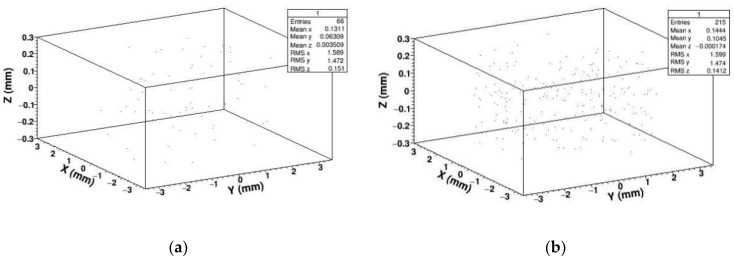
PKA spatial distribution in SDD generated by gammas: (**a**) PKA spatial distribution for 14 MeV gammas; (**b**) PKA spatial distribution for 20 MeV gammas.

**Figure 16:**
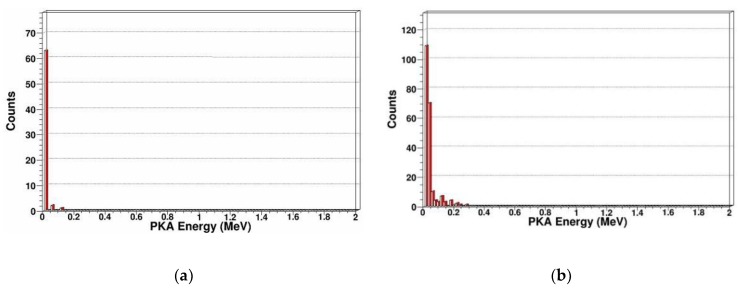
PKA energy spectrum in SDD generated by gammas: (**a**) PKA energy spectrum for 14 MeV gammas; (**b**) PKA energy spectrum for 20 MeV gammas.

In order to analyze the displacement damage caused by gamma photons, we reconstructed the spatial coordinates of PKAs in three dimensions and extracted the energy spectrum of PKA as shown in Figure 15 and Figure 16.

By three-dimensional coordinate reconstruction of PKA, we can see the number of PKA is very low. The number of recoil atoms generated by gamma photons which compare with neutrons is essentially negligible. This is because the probability of gamma photons producing PKAs in SDD is relatively small. However, gamma photons produce a large number of high-energy electrons in SDD. At the same time, the high-energy electrons can produce PKA in SDD. But the probability that high-energy electrons interact with lattice atoms in the SDD to produce PKA is very low, so the displacement damage generated by gamma photons is basically negligible.

- Secondary knock-on atom

**Figure 17:**
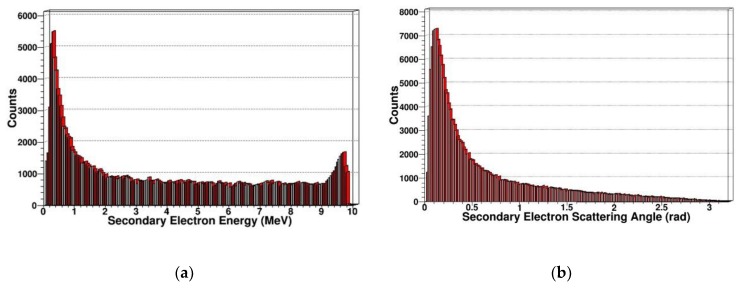
Secondary electrons in SDD generated by 14 MeV gammas: (**a**) Energy spectrum of secondary electrons; (**b**) scattering angle distribution of secondary electrons.

**Figure 18:**
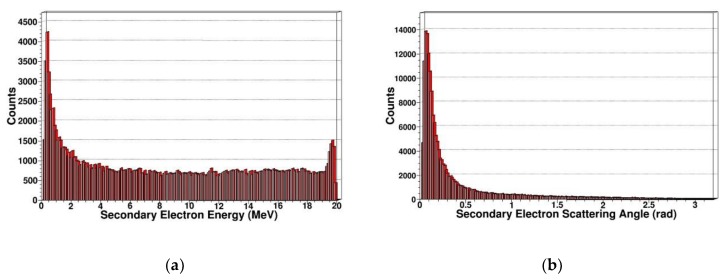
Secondary electrons in SDD generated by 20 MeV gammas: (**a**) Energy spectrum of secondary electrons; (**b**) scattering angle distribution of secondary electrons.

Since gamma photon mainly causes ionization damage, the ionization energy loss generated by gamma photons is an important factor leading to the radiation damage of SDD. Therefore, we simulated and extracted the energy spectrum and scattering angle distribution of secondary electrons generated by gamma photons as shown in Figure 17 and Figure 18. 

From the above results, we can see that for secondary electrons, the energy spectrum range is relatively wide, ranging from a few eV to the energy of incident particles, and many high-energy electrons are produced. This is consistent with the various effects of gamma photons in SDD, which can produce many electrons in the photoelectric effect, Compton effect, and pair production. When the Compton effect occurs, scattered photons can scatter in all directions. For scattered photons in different directions, the corresponding recoil electron energy is also different. Thus, even if the energy of the incident gamma photons is single, the energy of the recoil electrons continuously changes with the scattering angle, which can interpret the trend of energy spectrum of secondary electrons.

For the scattering angle distribution of secondary electrons, the scattering angle generated by gamma photons is distributed in 0–180 degrees, and the number of secondary electrons decreases as the angle increases, which corresponds to the tendency of the reaction cross section to decrease as the scattering angle increases in Klein–Nishina formula. This is basically consistent with the track structure produced by the gamma photons-irradiated SDD.

#### 3.3.2. Relationship between IEL and Thickness of SDD

The geometric size of SDD was changed by changing the thickness of substrate. The ionization energy deposition produced by 1 MeV gamma photons in SDD was recoded and fitted. The thickness of SDD is 100 μm, 200 μm, 300 μm, 400 μm, 500 μm, 600 μm, 700 μm, 800 μm, 900 μm, and 1000 μm.

**Figure 19:**
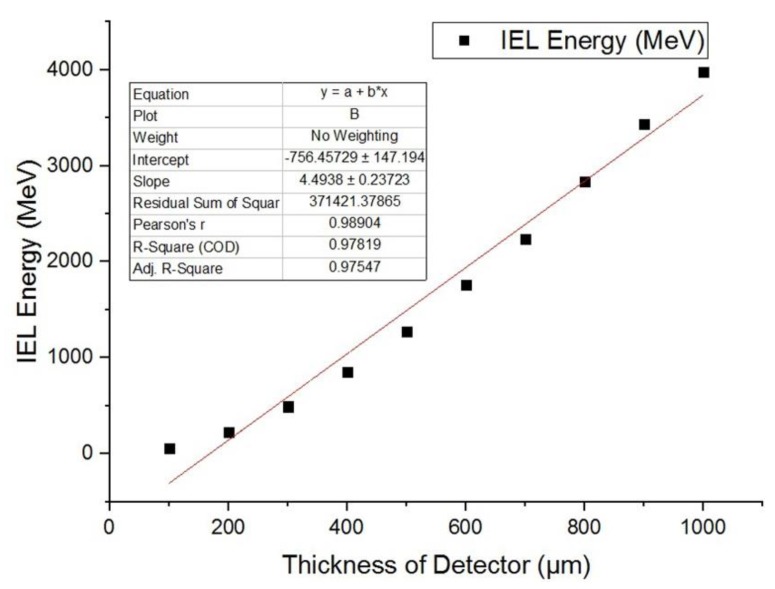
Relationship between IEL produced by gammas and the thickness of SDD.

As shown in Figure 19, we can see the ionization energy deposition produced by gamma photons in SDD is approximately linear with the thickness of SDD. Our simulation results indicate that on the micrometer scale, the ionization damage caused by gamma photons in SDD is approximately evenly distributed. Fitting the IEL curve in the above figure, we can obtain the coordinate of intersection between the curve and axis of detector thickness, which is (129.10, 0) and not (0, 0); this is not because of the simulation error, but rather implies when the detector thickness is lower than 129.10 μm, the IEL of gamma rays in SDD is 0. Therefore, considering both anti-irradiation reinforcement design and detection property of SDD, it is necessary to design a reasonable detector epitaxial thickness, which can capture the energy deposition of the target particle and reduce radiation damage caused by gamma rays.

Therefore, in the anti-radiation reinforcement design of SDD, it is necessary to rationally design the epitaxial thickness of the detector, which can reduce the ionization damage caused by gamma photons to SDD.

## 4. Conclusions

In this paper, we investigated the SDD radiation damage effects and mechanics for irradiations of different energy of neutrons and gammas using Geant4 simulation and obtained the effects of the displacement damage and ionization damage for SDD. Our research results indicate that gammas mainly cause the ionization damage in SDD, while neutrons induce the displacement damage and ionization damage in SDD. The distribution of displacement damage caused by neutrons is uniform. The ionization energy deposition and non-ionization energy deposition caused by neutrons in SDD are linear with the detector thickness. We simulated and analyzed the recoil atom energy spectrum, and results indicate that the recoil atoms are mainly concentrated in the low-energy region and are mainly due to low-energy transmission events. We also simulated the distribution of recoil atom scattering-angle distribution; the scattering angles are concentrated at 0 to 90 degrees, which shows that neutrons only have a collision in SDD. The energy deposition caused by gammas is linear with the thickness of SDD. Gammas generated a lot of secondary electrons in SDD, and the energy of secondary electrons is distributed from several eV to incident particle energy. As the scattering angle of secondary electrons increases, the number of secondary electrons decreases. Our research results will provide a scientific basis and theoretical support for SDD design and irradiation reinforcement applied in the field of deep space detection.

## References

[1] Pines D.J. (2004). ARPA/DARPA space programs. XNAV Ind. Day.

[2] Deng N.Q., Zhao B.S., Sheng L.Z. (2007). System of space voice communication based on X-ray. Acta Phys. Sin..

[3] (2015). Nasa Space Technology Roadmaps. https://www.nasa.gov/offices/oct/home/roadmaps/index.html.

[4] Gatti E., Rehak P. (1984). Semiconductor drift chamber-An application of a novel charge transport scheme. Nucl. Instrum. Methods Phys. Res..

[5] Nomerotski A. (2009). Silicon detectors for tracking and vertexing. Nucl. Instrum. Methods Phys. Res. Sect. A Accel. Spectrometers Detect. Assoc. Equip..

[6] Evangelista Y., Ambro F., Feroci M. (2018). Characterization of a novel pixelated silicon drift detector (PixDD) for high-throughput X-ray astrophysics. J. Instrum..

[7] Butt A.D., Fiorini C., Beretta M. (2018). Application of silicon drift detectors for the readout of a CdWO_4_ scintillating crystal. IEEE Trans. Nucl. Sci..

[8] Shanmugam M., Acharya Y.B., Vadawale S.V. (2016). Experimental characterization of silicon drift detector for X-ray spectrometry: Comparison with theoretical estimation. Measurement..

[9] Monte E.D., Rachevski A., Zampa G. (2014). Measurement of the effect of non ionizing energy losses on the leakage current of silicon drift detector prototypes for the LOFT satellite. J. Instrum..

[10] Monte E.D., Evangelista Y., Bozzo E. (2015). The effect of the displacement damage on the charge collection efficiency in silicon drift detectors for the LOFT satellite. J. Instrum..

[11] Harper R.S., Buttar C.M., Allport P.P. (2002). Evolution of silicon microstrip detector currents during proton irradiation at the CERN PS. Nucl. Instrum. Methods Phys. Res. Sect. A Accel. Spectrometers Detect. Assoc. Equip..

[12] Kramberger G., Cindro V., Mandic I. (2002). Effective trapping time of electrons and holes in different silicon materials irradiated with neutrons, protons and pions. Nucl. Instrum. Methods Phys. Res. Sect. A Accel. Spectrometers Detect. Assoc. Equip..

[13] Hayashi K., Park I., Dotsu K. (2013). Radiation effects on the silicon semiconductor detectors for the ASTRO-H mission. Nucl. Instrum. Methods Phys. Res. Sect. A Accel. Spectrometers Detect. Assoc. Equip..

[14] Pandey S.U., Vikelis G., Humanic T.J. (1995). Studies of ionizing radiation effects on silicon drift detectors. Nucl. Instrum. Methods Phys. Res. Sect. A Accel. Spectrometers Detect. Assoc. Equip..

[15] Kraner H.W., Li Z., Posnecker K.U. (1989). Fast neutron damage in silicon detectors. Nucl. Instrum. Methods Phys. Res. Sect. A Accel. Spectrometers Detect. Assoc. Equip..

[16] Li Z., Chen W., Dou L. (1992). Study of the long term stability of the effective concentration of ionized space charges N_eff_ of neutron irradiated silicon detectors fabricated by various thermal oxidation processes. IEEE Trans. Nucl. Sci..

[17] Mueller G.P., Wilsey N.D. (1982). The structure of displacement cascades in silicon. IEEE Trans. Nucl. Sci..

[18] Donegani E.M., Fretwurst E. (2018). Study of point- and cluster-defects in radiation-damaged silicon. Nucl. Instrum. Methods Phys. Res. Sect. A Accel. Spectrometers Detect. Assoc. Equip..

[19] Borodin V.A. (2012). Molecular dynamics simulation of annealing of post-ballistic cascade remnants in silicon. Nucl. Instrum. Methods Phys. Res. Sect. B Beam Interact. Mater. Atoms.

[20] Ruzin A., Casse G., Glaser A. (1999). Comparison of radiation damage in silicon induced by proton and neutron irradiation. IEEE Trans. Nucl. Sci..

[21] Saha U., Devan K. (2018). The computation of displacement damage cross section of silicon, carbon and silicon carbide for high energy application. Mater. Today Proc..

[22] Moll M., Feick H., Fretwurst E. (1997). Comparison of defects produced by fast neutrons and 60Co-gammas in high-resistivity silicon detectors using deep-level transient spectroscopy. Nucl. Instrum. Methods Phys. Res. Sect. A Accel. Spectrometers Detect. Assoc. Equip..

[23] Raine M., Jay A., Richard N. (2017). Simulation of single particle displacement damage in silicon-Part I; Global approach and primary interaction simulation. IEEE Trans. Nucl. Sci..

[24] GEANT4, a Simulation Toolkit. http://www.geant4.org/geant4.

[25] Lazanu I., Lazanu S. (2005). Silicon detectors: From radiation hard devices operating beyond LHC conditions to characterization of primary fourfold coordinated vacancy defects. Rom. Rep. Phys..

[26] Robinson M.T., Torrens I.M. (1974). Computer simulation of atomic-displacement cascades in solids in the binary-collision approximation. Phys. Rev. B.

